# Supplementary material for: The Role of Polymeric Coatings for a Safe-by-Design Development of Biomedical Gold Nanoparticles Assessed in Zebrafish Embryo
Source: Nanomaterials (Basel). 2021 Apr 14;11(4):1004. doi: 10.3390/nano11041004 (PMC8070688; doi:10.3390/nano11041004)
Supplement: Supplementary file 1 [file nanomaterials-11-01004-s001.pdf]

# Supplementary Material

*Article*

# The role of polymeric coatings for a safe-by-design development of biomedical gold nanoparticles assessed in Zebrafish embryo

**Pamela Floris[1†], Stefania Garbujo[2†], Gabriele Rolla[2], Marco Giustra[2], Lucia Salvioni[2], Tiziano Catelani[3], Miriam Colombo[2], Paride Mantecca[1,4*], Luisa Fiandra[2,4*]**

[1]POLARIS Research Centre, Dept. Of Earth and Environmental Sciences, University of Milano- Bicocca, Milan, Italy;
[2]Department of Biotechnology and Biosciences, University of Milano-Bicocca, Milan, Italy;
[3]Microscopy Facility, University of Milano-Bicocca, Milan, Italy;
[4]Centro 3R (Inter-University Center for the Promotion of the 3Rs Principles in Teaching & Research), Italy

*Correspondence: luisa.fiandra@unimib.it, Tel.: +39-02-64483406; paride.mantecca@unimib.it, Tel.: +39-02-64482916
† equally contributed to the work

**Table S1** $HT_{50}$ calculated on embryos exposed to 0.001-1 nM of 6 or 15 nm GNP@PMA or GNP@PEG. hpf: hours post-fertilization; u: upper confidence limit; l: lower confidence limit; n.d.: not detectable

| | GNP@PMA 6 nm hpf (u-l) | GNP@PMA 15 nm hpf (u-l) | GNP@PEG 6 nm hpf (u-l) | GNP@PEG 15 nm hpf (u-l) |
|---|---|---|---|---|
| **Control** | 79.37 (76.90 - 83.02) | 79.56 (76.71 - 84) | 81.58 (77.64 - 85.56) | 83.06 (78.57 - 87.86) |
| **0.001 nM** | 81.51 (78.90 - 84.27) | 75.66 (73.20 - 82.63) | 85.19 (81.83 - 88.47) | 80.82 (76.09 - 85.60) |
| **0.01 nM** | 76.26 (74.02 - 80.97) | 74.89 (72.57 - 82.74) | 92.13 (87.72 - 97.86) | 75.42 (69.59 - 81.07) |
| **0.1 nM** | 78.12 (75.68 -82.17) | 74.40 (72.09 - 83.80) | 87.66 (83.17 - 91.63) | 78.03 (71.14 - 84.93) |
| **1 nM** | 74.74 (72.66 - 80.66) | 76.09 (n.d.) | 86.13 (82.08 - 90.16) | 84.13 (76.89 - 92.02) |

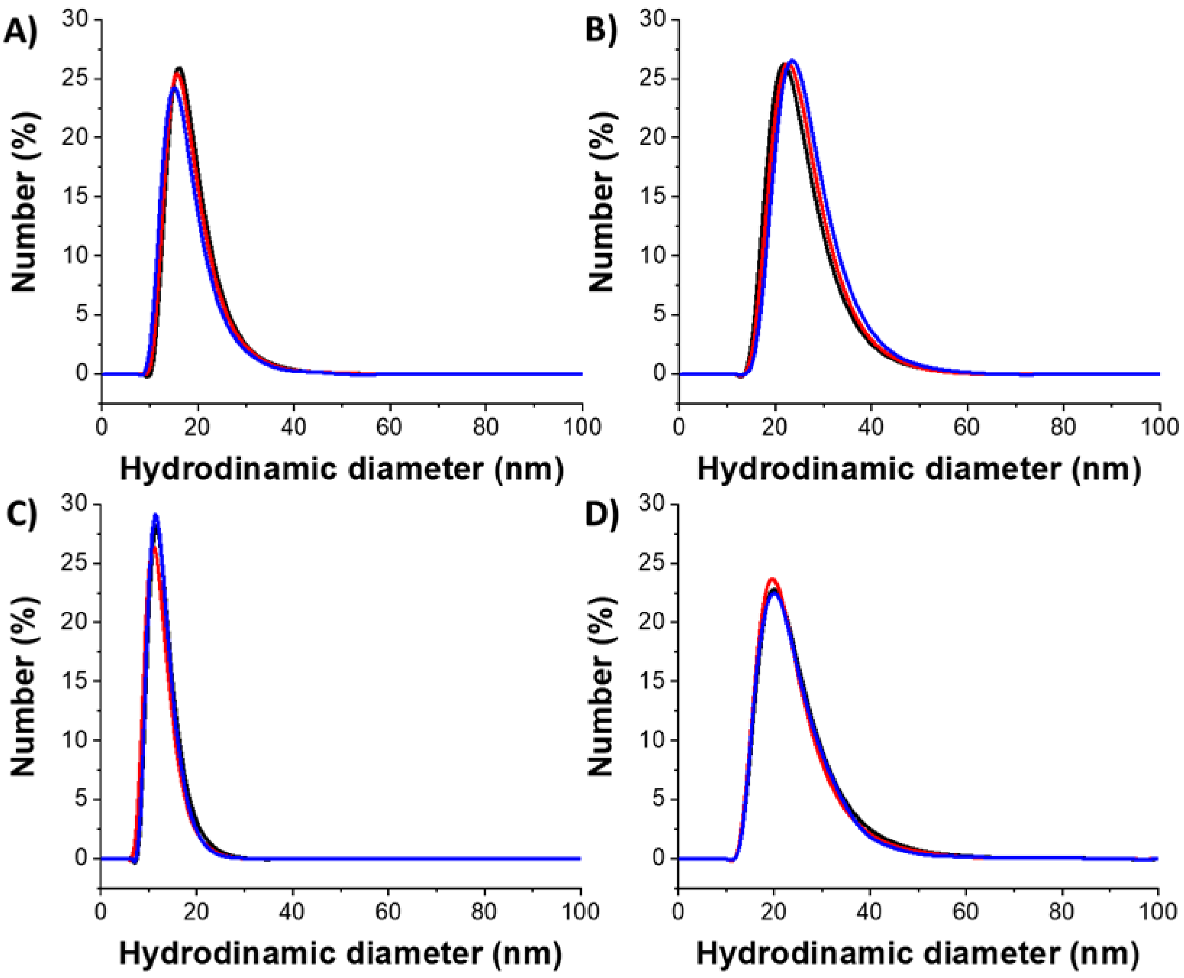


**Figure S1.** Distribution of hydrodynamic diameter about GNP measured by Dynamic Light Scattering of: A) 6 nm GNP@PEG, B) 15 nm GNP@PEG, C) 6 nm GNP@PMA, D) 15 nm GNP@PMA

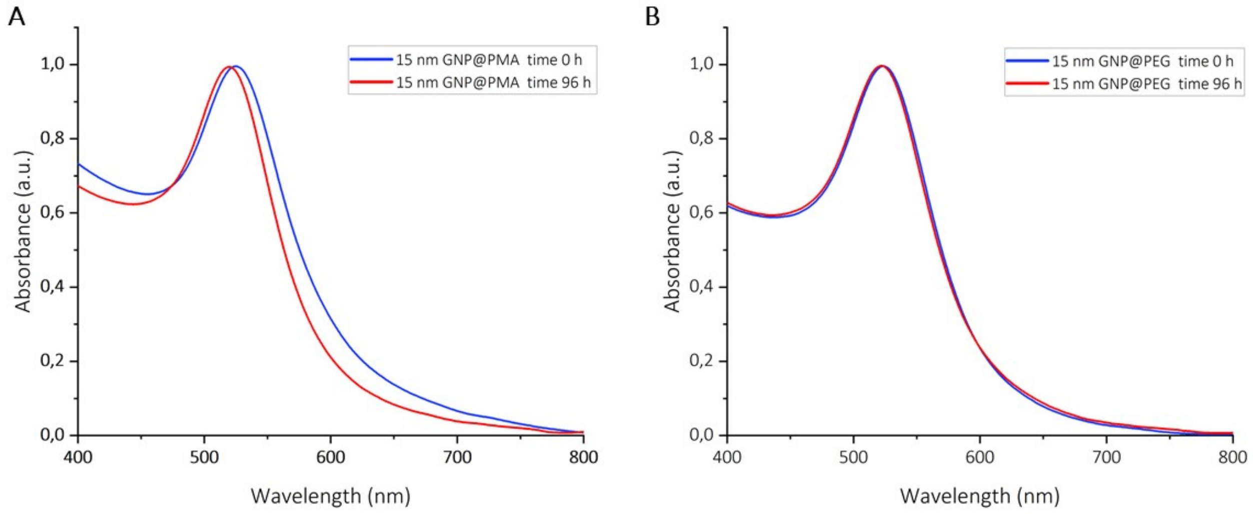


**Figure S2.** Stability of 15 nm GNP@PMA (A) and 15 nm GNP@PEG (B) in embryos solution was verified by UV-vis spectroscopy analysis immediately after their dilution (T= 0h) and after 96 hours of incubation (T= 96h).

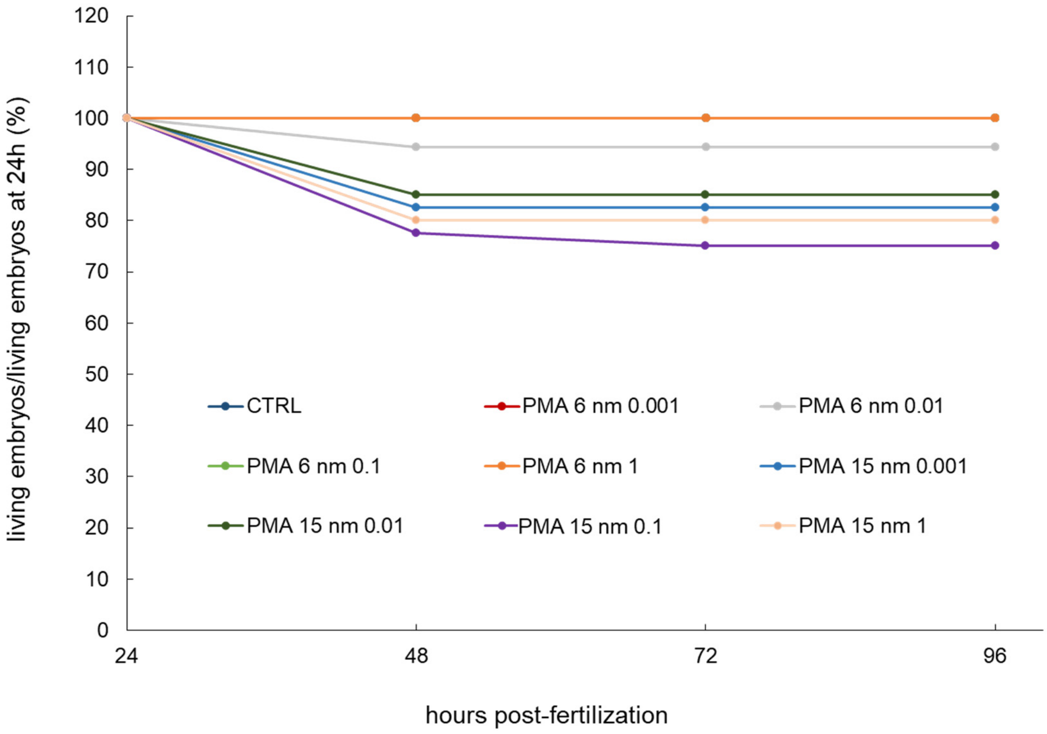


**Figure S3.** % of living embryos at 48, 72 and 96 hpf, after dechorionation (at 24 hpf) and exposure for to 6 nm or 15 nm GNP@PMA. Viability % has been calculated on the living embryos at 24 hpf. Embryos' viability values for CTRL, and for 6 nm GNP@PMA 0.001 and 0.1 nM, are all correspondent to 100% and therefore not visible into the graph.
